# The parasporal crystals of *Bacillus pumilus* strain 15.1: a potential virulence factor?

**DOI:** 10.1111/1751-7915.12771

**Published:** 2017-10-12

**Authors:** Diana C. Garcia‐Ramon, Colin Berry, Carmen Tse, Alberto Fernández‐Fernández, Antonio Osuna, Susana Vílchez

**Affiliations:** ^1^ Institute of Biotechnology Campus Fuentenueva University of Granada Granada Spain; ^2^ Cardiff School of Biosciences Cardiff University Cardiff UK; ^3^ Department of Biochemistry and Molecular Biology I Campus Fuentenueva University of Granada Granada Spain; ^4^ Present address: Medical School Faculty of Life, Health and Medical Sciences Universidad Internacional del Ecuador Quito Ecuador

## Abstract

*Bacillus pumilus* strain 15.1 was previously found to cause larval mortality in the Med‐fly *Ceratitis capitata* and was shown to produce crystals in association with the spore. As parasporal crystals are well‐known as invertebrate‐active toxins in entomopathogenic bacteria such as *Bacillus thuringiensis* (Cry and Cyt toxins) and *Lysinibacillus sphaericus* (Bin and Cry toxins), the *B. pumilus* crystals were characterized. The crystals were composed of a 45 kDa protein that was identified as an oxalate decarboxylase by peptide mass fingerprinting, N‐terminal sequencing and by comparison with the genome sequence of strain 15.1. Synthesis of crystals by a plasmid‐cured derivative of strain 15.1 (produced using a novel curing strategy), demonstrated that the oxalate decarboxylase was encoded chromosomally. Crystals spontaneously solubilized when kept at low temperatures, and the protein produced was resistant to trypsin treatment. The insoluble crystals produced by *B. pumilus* 15.1 did not show significant toxicity when bioassayed against *C. capitata* larvae, but once the OxdD protein was solubilized, an increase of toxicity was observed. We also demonstrate that the OxdD present in the crystals has oxalate decarboxylate activity as the formation of formate was detected, which suggests a possible mechanism for *B. pumilus* 15.1 activity. To our knowledge, the characterization of the *B. pumilus* crystals as oxalate decarboxylase is the first report of the natural production of parasporal inclusions of an enzyme.

## Introduction

The production of spore‐associated (parasporal) crystals by several species of bacteria within the genus *Bacillus* and related genera is well known. These proteins are almost always entomopathogenic toxins, active against a wide range of invertebrate targets (Bechtel and Bulla, 1976; Bravo *et al*., 2007), although crystals without known targets (sometimes termed parasporins) are also known. Such parasporins are related in sequence and structure to known invertebrate‐active toxins, and it is likely that their natural target merely remains to be discovered (although activity against certain human cancer cells in culture has been reported (Ohba *et al*., 2009)). The most studied proteinaceous toxins are the Cry and Cyt toxins, produced mainly by *Bacillus thuringiensis* (*Bt*), which are the principal agents responsible for the toxicity of these bacteria towards insects. The insecticidal activity of crystal proteins produced by *Bt* has been extensively used as the basis of many commercial products. The ability to produce parasporal crystals is not restricted to *Bt* as some strains of *Lysinibacillus sphaericus* (Jones *et al*., 2007), *Clostridium bifermentans* (Barloy *et al*., 1996), *Paenibacillus popilliae* (Zhang *et al*., 1997), *Brevibacillus laterosporus* (Smirnova *et al*., 1996) and *P. lentimorbus* (Yokoyama *et al*., 2004), also produce parasporal inclusions active against insects.

The mechanisms of action proposed for the parasporal crystal toxins generally require solubilization and proteolytic activation of the protoxin form in the midgut of the target invertebrate (Haider *et al*., 1986; Palma *et al*., 2014). Serine proteases are important in both solubilization and activation of *Bt* protoxins and, in some insects, changes in the protease profile of their guts have been associated with resistance to *Bt* toxin (Li *et al*., 2004; Karumbaiah *et al*., 2007).

Our research group reported a *Bacillus pumilus* strain toxic towards the Mediterranean fruit fly, *Ceratitis capitata* (Molina *et al*., 2010). Previous assays showed that the toxicity of *B. pumilus* 15.1 can be inactivated either by heat or by proteases, suggesting that the virulence factor produced by this strain could be proteinaceous (Molina, 2010). As its initial isolation and testing, our strain appears to have decreased in toxicity, even though it continues to produce the parasporal crystals, mainly composed of a 45 kDa protein, that we have previously described (Garcia‐Ramon *et al*., 2016). Despite the loss of toxicity, the crystal protein was still considered as a candidate toxin (possibly interacting with another factor, now lost or under‐expressed). In this work, we describe the characterization of the crystal inclusions produced by *B. pumilus* 15.1 as the first example of a parasporal enzyme crystal, and we propose a potential mechanism of action for this entomopathogenic strain.

## Results

### Identification of the crystal protein

The spore‐crystal complex of a *B. pumilus* 15.1 culture, sporulated in T3 medium, was used to isolate crystals using sucrose density gradient centrifugation (Garcia‐Ramon *et al*., 2016). Crystals formed bands at the interface formed between the solutions of 72% and 79% sucrose (like many Cry toxins (Thomas and Ellar, 1983; Koller *et al*., 1992; Jones *et al*., 2007; Swiecicka *et al*., 2008)). They were also found at the 79%/84% sucrose interface. The enriched crystal proteins from both bands produced a major band of 45 kDa on SDS‐PAGE, as previously observed (Garcia‐Ramon *et al*., 2016), and this was excised for fingerprint analysis using MALDI‐TOF MS. Mass spectrometry of the intact protein revealed a mass of 43 799 Da. Comparison of mass peaks obtained from fingerprinting with the recently published *B. pumilus* 15.1 genome (Garcia‐Ramon *et al*., 2015a) and *Bacillus* databases produced matches (40.8% sequence coverage) with OxdD, a putative oxalate decarboxylase encoded in Contig 4 of the *B. pumilus* 15.1 strain genome and an OxdD from *B. pumilus* ATCC 7061. The predicted MW of this *B. pumilus* 15.1 OxdD protein was 43 799.1 Da, corresponding to the molecular weight determined by MS. The N‐terminal analysis of this ~45 kDa protein, after treatment with trypsin (see below), rendered the sequence S‐E‐K‐P‐D/N‐G‐I‐P. The SEKPNGIP sequence showed 100% identity and 100% sequence coverage with oxalate decarboxylase from *B. pumilus* 15.1 (accession number KLL01117) and other *B. pumilus* strains (KIL13977) from their second amino acid (the initiator methionine was missing as frequently occurs with *in vivo* methionine aminopeptidase activity, particularly when the next residue is small, such as the Ser residue in this case (Xiao *et al*., 2010).

**Figure 1:**
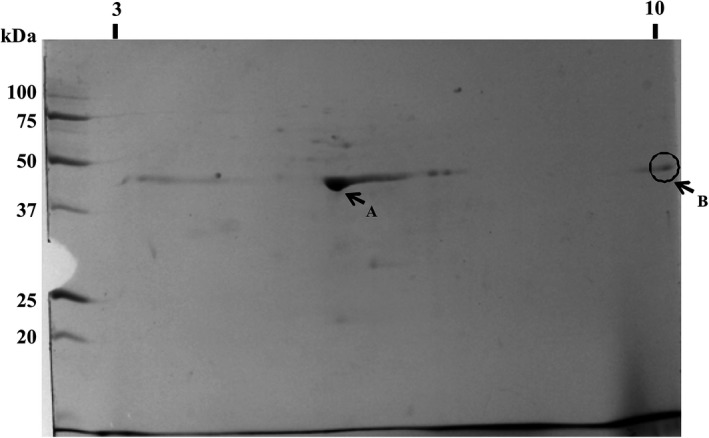
Two‐dimensional electrophoresis of a fraction obtained from the sucrose gradient of a *B. pumilus* 15.1 culture. The pH (p*I*) range is shown horizontally, and molecular weight (kDa) is shown vertically. The p*I* ranged from 3 to 10. Arrow A shows p*I* 5.5; Arrow B shows p*I* ≥ 10.

The 45 kDa protein was also subjected to 2D electrophoresis for protein characterization (Fig. 1), and two spots at approximately p*I* 5.5 (spot A) and p*I* 10 (spot B) were observed. Both spots were analysed by MALDI‐TOF MS and identified as oxalate decarboxylase. The theoretical p*I* of oxalate decarboxylase is 5.22, which corresponds with the p*I* observed for spot A. The appearance of spot B at p*I* 10 is unexplained as this does not fit with the theoretical value and, as far as we know, there is no reported oxalate decarboxylase with a p*I* ≥ 10 in the literature.

**Figure 2:**
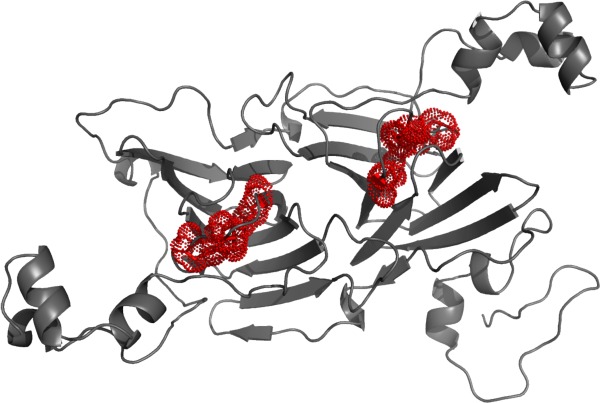
OxdD model. The model of *B. pumilus* 15.1 OxdD was produced using Swiss model. The two conserved Mn^2+^ binding sites (H96, H98, E102 and H274, H276, E281) are coloured red and shown with sticks and dots. The symmetry of the molecule with its two cupin domains (left and right) can be seen clearly.

Taken together, the data above indicate that the 45 kDa protein is that encoded by the *B. pumilus* strain 15.1 *oxdD* gene and the protein will be described from this point as oxalate decarboxylase. Features of this protein family, including the two Mn^2+^ binding sites, are conserved in the *B. pumilus* protein, and we were able to construct a molecular model of the protein based on the known structure of the *B. subtilis* enzyme (PDB accession 5HI0) using the Swiss model program (Schwede *et al*., 2003) as shown in Fig. 2.

### The *oxdD* gene

**Figure 3:**
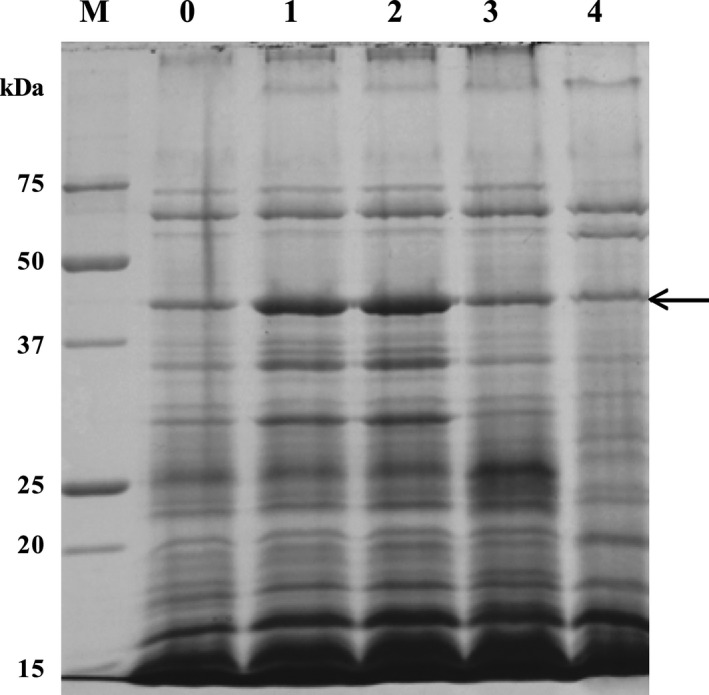
Protein profile of the pellet fractions of *B. pumilus* 15.1 cultures grown on T3 medium in the presence of different concentrations of MnCl_2_. The standard conditions for MnCl_2_ were 5 mg l^−1^ (lane 2). Lane 0 shows a pellet fraction of a culture without MnCl_2_, lane 1 with 0.5 mg l^−1^ MnCl_2_, lane 3 with 50 mg l^−1^ MnCl_2_ and lane 4 with 0.5 g l^−1^ MnCl_2_. Lane M shows a molecular weight marker (Precision Plus Bio‐Rad) in kDa. The arrow shows the oxalate decarboxylase protein.

The *oxdD* gene is located on Contig 4 of the draft *B. pumilus* 15.1 genome. Analysis of the region upstream of this gene using the DBTBS database of transcription factors in *B. subtilis* (Sierro *et al*., 2008) predicts that the gene is preceded by a putative sigma K promoter (GGCCTTTTGTCACCTCACACCATACGATG) beginning 47 nt upstream of the initiator ATG. Regulation by this late mother cell sigma factor would be consistent with previous studies that demonstrated that *B. pumilus* strain 15.1 produces the crystal protein during sporulation when cultured in T3 medium, showing a maximal accumulation after 72 h (Garcia‐Ramon *et al*., 2016). Sigma K is also used in the production of some Cry proteins in *Bt* (reviewed in (Deng *et al*., 2014)). In addition, beginning 80 nt upstream of the ATG is a putative MntR transcription factor site (GTTTCACCTTATGAAAACG). This site is normally associated with regulation of Mn^2+^ transport with repression of *mntH* at high Mn^2+^ concentrations. The Mn^2+^ ion is the only trace element present in T3 medium with a standard concentration of 5 mg l^−1^ of MnCl_2_•4H_2_O (25 μM), so we analysed the accumulation of oxalate decarboxylase at concentrations ranging from 0 to 0.5 g l^−1^ (0 to 2.5 mM). The cultures all reached comparable cell densities at the end of the incubation period and the results showed (Fig. 3) that oxalate decarboxylase was present at all Mn^2+^ concentrations tested, showing maximal accumulation at 0.5 and 5 mg l^−1^ of MnCl_2_ (Fig. 3, lanes 1 and 2). The variation of oxalate decarboxylase seen in these experiments may be due to variations in expression, possibly mediated via the putative MntR region. Alternatively, the stability of the oxalate decarboxylase could also be involved because the Mn^2+^ binding sites are conserved in the *B. pumilus* 15.1 protein (Fig. 2). However, we might expect stabilization to be greater at higher Mn^2+^ concentrations, which is the opposite the effect seen in our experiments.

### The oxalate decarboxylase protein shows unexpected solubilization behaviour

**Figure 4:**
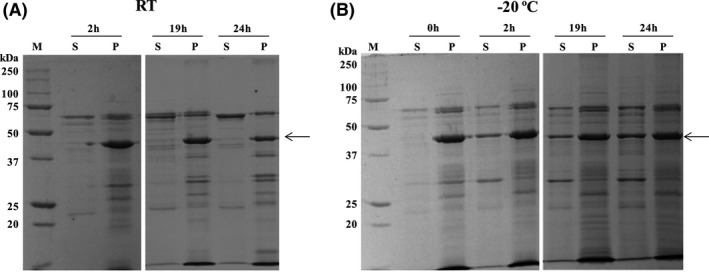
SDS‐PAGE analysis of the pellet and supernatant fraction of oxalate decarboxylase crystals obtained from a fresh sucrose gradient and kept at room temperature RT (panel A) or low temperature (Panel B). The incubation at −20°C solubilized the 45 kDa oxalate decarboxylase over time while during incubation at RT the protein remained in the insoluble fraction. Lanes S represent the supernatant fractions, and lanes P represent the pellet fractions of the samples. The arrows indicate the oxalate decarboxylase protein. Lanes M show the Precision Plus Bio‐Rad molecular weight marker in kDa.

When protein crystals are formed, subsequent solubilization can be expected to occur to release their potential (as occurs with crystal toxins). The crystal toxins of Bt often solubilize at pH values ≥ 9.0, so solubility of the oxalate decarboxylase crystals was tested under similar conditions. In our standard procedure, crystals from the sucrose gradient, washed with PBS, were resuspended in milliQ water and kept at −20°C. When crystals were used, an aliquot of the thawed crystal suspension was centrifuged, the supernatant was discarded and the pellet resuspended for 1 h at 37°C in 0.1 M sodium phosphate pH 9.0. After this time, samples were centrifuged, and soluble and insoluble fractions were analysed by SDS PAGE. Approximately 50% of the crystal protein was solubilized at pH 9.0 (results not shown), but total protein content (soluble and insoluble) was considerably lower than expected. Reanalysis of the stored sample revealed that the protein content of the crystals kept at −20°C (pellet fraction) decreased over time, with the crystals of oxalate decarboxylase protein becoming solubilized into the supernatant fraction on low temperature storage. To verify this phenomenon, a fresh crystal preparation was divided into two fractions. One was kept at −20°C and the second at room temperature (RT). Ten microlitre samples were taken over time from each aliquot, centrifuged, pellet and supernatant separated, and analysed by SDS‐PAGE gels. The results presented in Fig. 4 showed that when the crystal preparation was kept at RT the oxalate decarboxylase was observed only in the pellet fractions (Fig. 4A). In contrast, when the sample was kept at −20°C, the concentration of oxalate decarboxylase in the supernatant fraction increased as the incubation time at −20°C progressed (Fig. 4B). Transmission electron microscopic analysis of crystals revealed that the sample incubated at RT‐contained parasporal crystals, while the sample incubated at −20°C (for longer than 24 h) showed almost no crystals at all (data not shown). The protein from the pellet and supernatant fractions obtained after incubation at −20°C was identified by MALDI‐TOF MS and LC‐MS/MS (soluble fraction only) to rule out the possibility that other proteins may have been present in the crystals. Once again, MALDI‐TOF results identified only oxalate decarboxylase, in the pellet (36% coverage) and in the supernatant (34% coverage). Size‐exclusion chromatography of the soluble protein indicates that the protein exists in solution in a multimeric form with the protein eluting from the column at a volume, compared with molecular weight standards, consistent with a hexameric assembly (Fig. S1).

### The oxalate decarboxylase protein is resistant to trypsin

**Figure 5:**
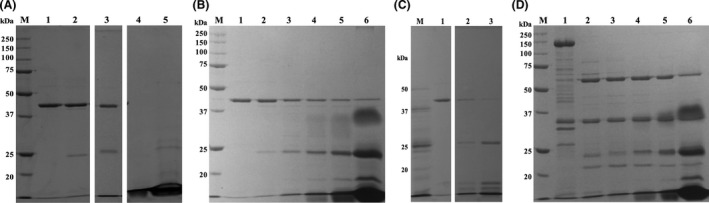
SDS‐PAGE analysis of the oxalate decarboxylase and the Cry1Aa13 digested with different proteases. Panel A shows the oxalate decarboxylase digested with trypsin (lane 2), chymotrypsin (lane 3), papain (lane 4) and ‘proteinase from *B. subtilis’* (lane 5). Panel B and C shows digestions of the oxalate decarboxylase with trypsin (Panel B) and chymotrypsin (Panel C) at protein:protease ratios 1:1 (lanes 2), 1:10 (lanes 3), 1:50 (lanes 4), 1:100 (lanes 5) and 1:500 (lanes 6). Panel D shows the digestion of Cry1Aa13 at the same protein:protease ratios as Panel B. As control, lanes 1 show the soluble proteins with no protease treatment. Lanes M show the molecular mass marker (Precision Plus Bio‐Rad) in kDa.

The soluble protein (obtained by incubation of the crystals at −20°C) was digested with a range of proteases to determine whether the protein was susceptible to their action or was (partially) resistant (as would be expected, e.g. for Cry toxins). Trypsin, chymotrypsin, papain and ‘Proteinase from *B. subtilis’* were tested at a 10:1 ratio (w/w) protein:enzyme. SDS‐PAGE analysis revealed that the oxalate decarboxylase protein was completely digested by papain and ‘Proteinase from *B. subtilis’* (Fig. 5, Panel A, lanes 4 and 5), while trypsin and chymotrypsin gave no visible digestion at this protein:enzyme ratio (Fig. 5, Panel A, lanes 2 and 3 respectively). Increasing the quantity of chymotrypsin (1:1 and 1:10 protein:enzyme), produced increasing degradation of the oxalate decarboxylase (Fig. 5, Panel C) but protein:trypsin ratios of 1:1 to 1:500 still produced no change in the band (Fig. 5, Panel B, lanes 2–6), while this enzyme was able to activate solubilized Cry1Aa13 used as control to produce the expected 66 kDa product (Fig. 5, Panel D).

### Investigating the location of the *oxdD* gene in *B. pumilus* 15.1

The majority of crystal toxin genes of *Bt* are encoded on extrachromosomal elements, and we decided to investigate the location of the *oxdD* gene. We have recently shown that the *B. pumilus* 15.1 strain bears one plasmid of 7785 bp named pBp15.1S (Contig 38) and one megaplasmid of unknown size named pBp15.1B (Garcia‐Ramon *et al*., 2015b). The *oxdD* gene was found in Contig 4 (Accesion number LBDK01000004), a contig of 57 329 bp that encodes 51 predicted proteins. This contig is distinct from the small plasmid pBp15.1S but has a size that could either represent part of a megaplasmid or a chromosome fragment. In order to determine if the crystals produced by *B. pumilus* 15.1 strain were encoded by the chromosome or the megaplasmid we decided to cure the strain of its extrachromosomal elements.

### Obtaining *B. pumilus* 15.1 variants without extrachromosomal elements

Different methodologies described in the literature for curing extrachromosomal elements such as heat and SDS treatment, acridine orange and promethazine treatment (detailed in the Materials and Methods section) were used without any success (data not shown).

**Figure 6:**
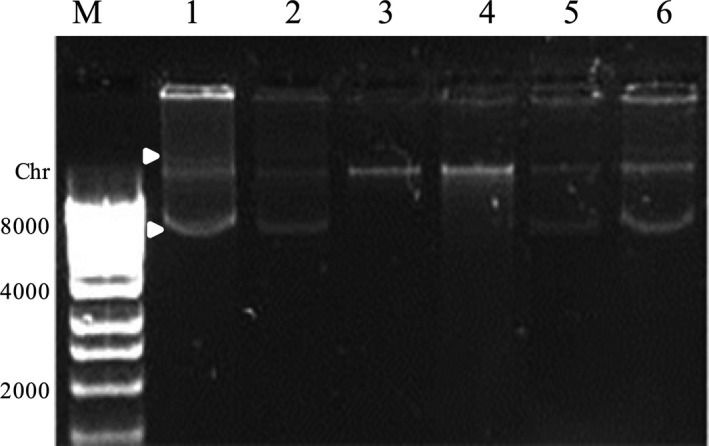
DNA electrophoresis in 0.8% agarose gel of total DNA extracted from several *B. pumilus* 15.1 variants. Wild‐type strain is shown in lanes 1 and 2. Variants obtained with the prior formation of spheroplasts are shown in lanes 3 (treated with acridine orange) and 4 (treated with promethazine). Lanes 5 and 6 show two variants treated with acridine orange and promethazine respectively without lysozyme treatment. M: Molecular weight marker (HyperLadder I from Bioline) in base pairs. White arrows indicate the megaplasmid (pBp15.1B) and the plasmid (pBp15.1S) respectively, and black arrow indicates the chromosomal DNA.

**Figure 7:**
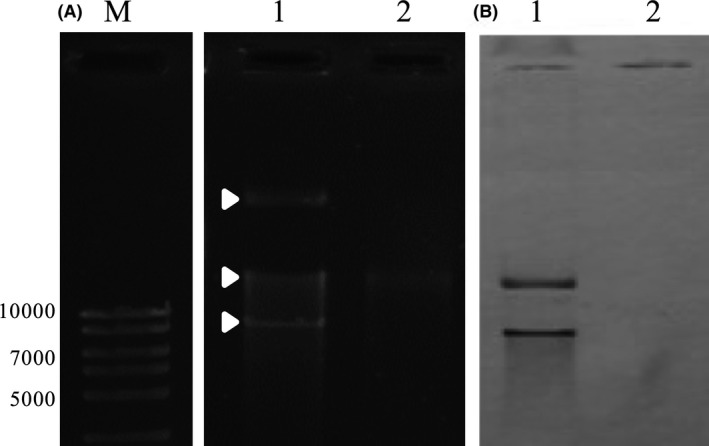
DNA electrophoresis (Panel A) and Southern blot (Panel B) of total DNA from *B. pumilus* 15.1 wild type (lanes 1) and *B. pumilus* 15.1C (lanes 2). Electrophoresis was performed in a 1% agarose gel and stained with ethidium bromide. Sothern blot was performed with a DIG‐labelled probe designed in the *orf*7 of the plasmid pBp15.1S (Garcia‐Ramon *et al*., 2015b). M: Molecular weight marker (HyperLadder I from Bioline) in base pairs. The white arrows indicate the megaplasmid, the chromosome and the plasmid from top to bottom respectively.

In a previous characterization of the *B. pumilus* 15.1 strain under electron microscopy (Garcia‐Ramon *et al*., 2016), we observed that the strain showed a particularly thick cell wall. We hypothesized that the lack of effect of the compounds tested for plasmid curing might be caused by the difficulty that these compounds might encounter in penetrating the cells to interfere with plasmid replication. For that reason, we designed a strategy to improve the success of compound internalization and hence the success of plasmid curing. The strategy consisted of obtaining spheroplasts from *B. pumilus* 15.1 with the use of lysozyme prior to the treatment with the replication‐interfering compounds. We tested our hypothesis with acridine orange and promethazine, two very well‐known curing compounds. *B. pumilus* 15.1 spheroplasts were obtained from vegetative cells as detailed in the Materials and Methods section, and then they were diluted in LB medium containing acridine orange (0.03%) or promethazine (0.12%). As controls, the same amount of vegetative cells, without the lysozyme treatment, were treated under the same conditions in the presence of the replication‐interfering compounds. When total DNA was extracted from one colony obtained from each treatment (Fig. 6) no extrachromosomal elements were observed in those cells previously treated with lysozyme (Fig. 6, lanes 3 and 4). In contrast, those cells not treated with lysozyme (Fig. 6, lanes 5 and 6) showed the presence of extrachromosomal elements in their cytoplasm. The use of the spheroplasts instead of the vegetative cells seems to improve the efficiency of acridine and promethazine in curing the strain *B. pumilus* 15.1. The acridine orange strain was selected for further studies and named *B. pumilus* 15.1C (cured from plasmid (pBp15.1S) and megaplasmid (pBp15.1B)). As, in contrast to the megaplasmid, the smaller pBp15.1S plasmid has been completely characterized, and its copy number was found to be 33 (Garcia‐Ramon *et al*., 2015b), we were able to verify its absence by PCR since, using the same methodology: no amplification was obtained from *B. pumilus* strain 15.1C (data not shown). Southern blot analysis using a Dig‐labelled probe designed in the *orf*7 of the plasmid pBp15.1S was also carried out. The probe hybridizes to the smaller band in the gel, corresponding to the small plasmid and also interacts with the chromosomal band, most likely due to entanglement of the plasmid with chromosomal DNA. No signal (for either band) was observed in the lane corresponding to total DNA from cured *B. pumilus* 15.1C (Fig. 7 Panel B, lane 2), verifying the absence of plasmid pBp15.1S.

### The gene encoding oxalate decarboxylase in *B. pumilus* 15.1 has a chromosomal location

**Figure 8:**
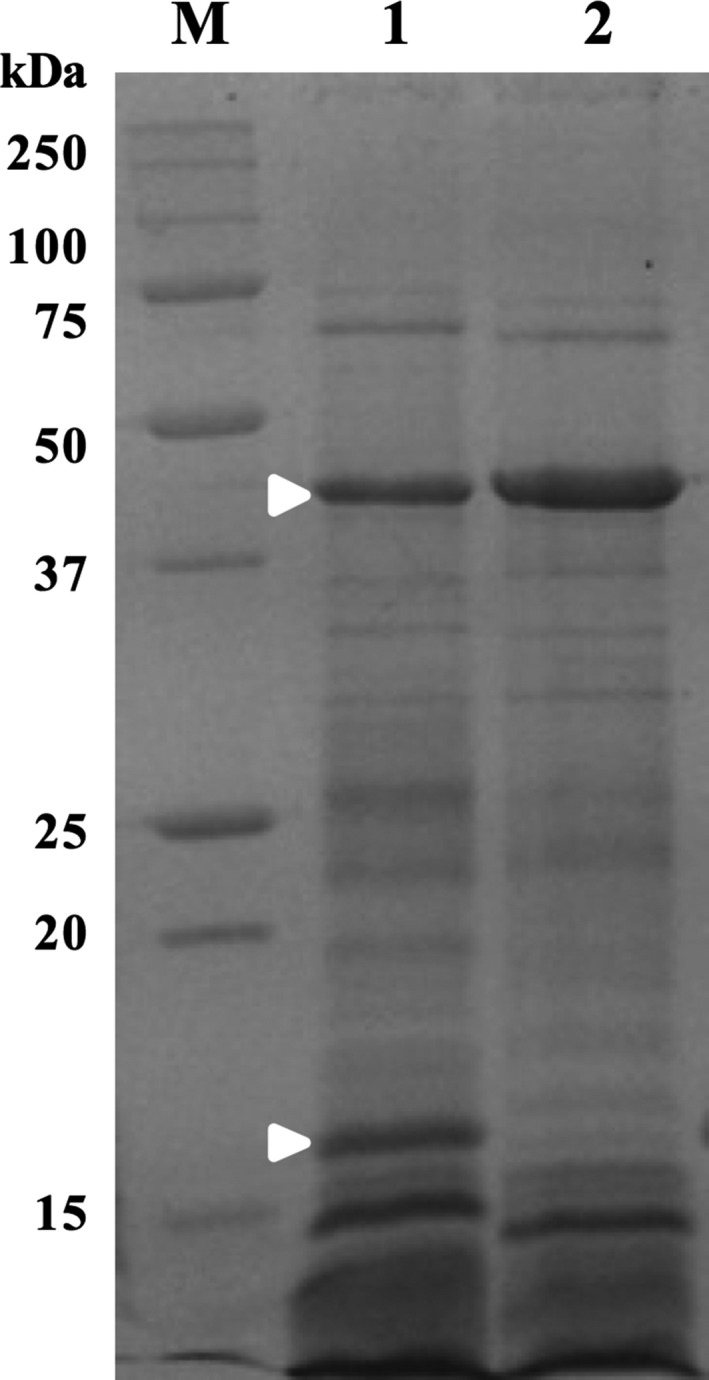
SDS‐PAGE analysis of the pellets from *B. pumilus* 15.1 and *B. pumilus* 15.1C cultures. White arrows show the oxalate decarboxylase protein at 45 kDa in the wild type (lane 1) which is more intense in the cured strain (lane 2) and the 17 kDa protein present only in the wild‐type strain. Lane M shows the molecular weight marker (Precision Plus Bio‐Rad) in kDa.

The protein profile of the pellet fraction of a 72‐h culture of the cured strain *B. pumilus* 15.1C was obtained, analysed by SDS‐PAGE and compared to the *B. pumilus* 15.1 protein profile previously described (Garcia‐Ramon *et al*., 2016). Although the general pattern of proteins was conserved, two main differences were observed: (i) the accumulation of the 45 kDa oxalate decarboxylase seems to be higher in the cured strain compared to the wild type (Fig. 8), and (ii) an approximately 17 kDa protein was missing in the cured strain compared to the wild‐type (Fig. 8, lower white arrow).

A MS fingerprinting analysis of the 17 kDa protein treated with trypsin produced two amino acid sequences (VLPAAGTYTFR and FYAEDTLDIQTRPVVVTPPDPCGC) both showing identity with the product of the *yuaB* gene from *B. pumilus* 15.1 localized in Contig 48 and with the hypothetical protein BPUM_1610 of *B. pumilus* SAFR‐032 (accession number ABV62292.1). The coverage of the sequence was around 19%, the predicted molecular weight of the 175 aa protein was 19 297 Da including a predicted signal peptide of 27 aa, the removal of which would yield a 16.3 kDa protein, consistent with the size observed in SDS PAGE gels. This protein shows 67% identity with the *Bacillus subtilis* BslA protein; a protein with an immunoglobulin‐like fold that forms a hydrophobic coat on biofilms (Hobley *et al*., 2013; Bromley *et al*., 2015).

Taking these results together, we can conclude that the oxalate decarboxylase of *B. pumilus* 15.1 is not encoded by the megaplasmid pBp15.1B, as it is expressed in the cured strain *B. pumilus* 15.1C, and, therefore, the *oxdD* gene is localized in the chromosome. We can also conclude that it is highly probable that the gene encoding the 17 kDa BslA‐like protein is present in the megaplamid pBp15.1B as the protein does not express in the cured strain. To prove this, two primers based on the gene *yuaB* in the strain 15.1 genome (Garcia‐Ramon *et al*., 2015a) were designed. A 727 bp product was detected only when DNA from the wild‐type strain was used as template, but not when total DNA from *B. pumilus* 15.1C was used (data not shown). As the *yuaB* gene is not present in the known sequence of pBp15.1S (Garcia‐Ramon *et al*., 2015b) and as the strain contains only one plasmid and one megaplasmid, we must conclude that *yuaB* gene is present in the megaplasmid pBp15.1B. The 24 079 bp Contig 48 (LBDK01000048), where the *yuaB* gene is present must therefore, be part of this megaplasmid and contains 27 CDSs, most of them encoding hypothetical proteins.

When *B. pumilus* 15.1C was analysed under transmission electron microscopy no morphological differences were observed compared to *B. pumilus* 15.1 strain (data not shown). The only remarkable difference was that the number of crystals in *B. pumilus* 15.1C cultures was higher than in *B. pumilus* 15.1. A quantification of the number of crystals and spores from different fields of the micrographs obtained, showed that the ratio crystals:spore observed in a culture of *B. pumilus* 15.1C was 0.17:1 compared to the ratio 0.09:1 previously determined for *B. pumilus* 15.1 (Garcia‐Ramon *et al*., 2016). This result seems to indicate that the production of the crystals in the cured strain was higher (almost double) than in the wild‐type strain, a fact, that is, in agreement with the observation from SDS‐PAGE that the expression of the oxalate decarboxylase protein is higher in the cured strain (Fig. 8).

### Purified and insoluble crystals produced by *B. pumilus* 15.1 are not toxic

The crystal bands from sucrose gradients obtained from the wild‐type *B. pumilus* 15.1, containing the majority of the oxalate decarboxylase, were tested in bioassays against first‐instar larvae of *C. capitata* using deionized water as negative control. As stated above, the activity of strain 15.1 has decreased since initial isolation, but it is possible that the purified crystal, assayed at high concentrations might produce an increase in toxicity. When biossayed (Table 1) crystals obtained from *B. pumilus* 15.1 showed a mortality of only 4.2% compared to that obtained in the negative control (6.25% mortality). We then tested the activity of the crystal fractions after being frozen at −20°C for 4 h to promote solubilization, performing bioassays with the pellet and supernatant separately. The pellet fraction of *B. pumilus* 15.1 caused 6.79% mortality, while supernatant caused 18.8%. In the negative control, where just water was bioassayed, a mortality of 2.08% was recorded. We observed that solubilized crystals were slightly more toxic (3 fold) than the non‐solubilized protein, even though a very short period of time for solubilization was allowed (only 4 h). These results may indicate that oxalate descarboxylase could be involved in toxicity, and it needs to be in a soluble form to exert its action.

**Table 1:** Mortality results obtained after 10 days in *C. capitata* larvae bioassays using insoluble and soluble crystals obtained from *Bp* 15.1 after sucrose gradient purification and incubation at −20°C for solubilization. The increase in toxicity compared to the negative control was also calculated

Bioassay	% Mortality	Fold increase
H_2_O (−ve control)	6.25 ± 2	1
Untreated crystals from *Bp*15.1	4.2 ± 1	0.6
Solubilized crystals from *Bp*15.1	18.8 ± 3	3.0
Pellet remaining after solubilization	6.79 ± 2	1.1

### Oxalate decarboxylase is enzymatically active and produces formate from oxalate

With the objective of demonstrating if the oxalate decarboxylase produced by *B. pumilus* 15.1 as inclusion crystals is enzymatically active, two different enzymatic assays were set‐up. In the first assay, the oxalate decarboxylase activity assay kit (Sigma Aldrich) was used to assay approximately 1 μg of solubilized crystal protein. The *B. pumilus* protein produced approximately 7 times more formate than the positive control enzyme (7 μl) provided with the kit (9.27 and 1.25 nmol formate respectively). In the second assay, *B. pumilus* 15.1 crystals were purified in a sucrose gradient, resuspended in Mili Q water, kept at −20°C for 96 h for solubilization and quantified by the Bradford method. Five or ten micrograms of soluble protein were included in the enzymatic assays using sodium oxalate as a substrate. The activity of the enzyme was evaluated in the presence and absence of Mn^2+^ (as this ion is a cofactor for the enzyme). After stopping the reaction, the production of formate was analysed by ^1^H‐NMR. For quantification purposes, 5 mM of methanol was added to each sample as an internal reference just before the ^1^H‐NMR spectra were obtained. The spectra are detailed in Fig. S2 Formate production was detected as a singlet at 8.40 ppm in all the spectra. After integrating the area of the formate peak and comparing with the area of the methanol signal (3.31 ppm), the concentration of formate was estimated (Table 2). Enzymatic assays containing 10 μg of the enzyme produced twice the amount of formate as those containing 5 μg of enzyme. When the enzyme was not included in the assay, formate was not detected (data not shown), ruling out the possibility of spontaneous decomposition of oxalate. Although Mn^2+^ is described to be cofactor for oxalate decarboxylase, the production of formate was significantly reduced (around 50%) when 1 mM of the ion was present in the enzymatic reaction.

**Table 2:** Integral value of peaks at 8.40 ppm (corresponding to formate) and estimated formate concentration using methanol as internal reference. Formate production was evaluated in the presence (1 mM) and the absence of Mn^2+^ ions and with different amounts of oxalate decarboxylase enzyme

μg of enzyme	−Mn^2+^		+ Mn^2+^	
	Integral value^a^	Formate^b^ concentration (mM)	Integral value^a^	Formate^b^ concentration (mM)
0	0	0	0	0
5	0.19 ± 0.00	0.31 ± 0.00	0.11 ± 0.00	0.18 ± 0.01
10	0.42 ± 0.01	0.60 ± 0.02	0.18 ± 0.00	0.29 ± 0.00

a Mean of the integral values obtained in two different enzymatic assays.

b Estimated formate concentration using 5 mM methanol as internal reference.

### Formate has an effect on the development of *C. capitata* larvae

After demonstrating that oxalate decarboxylase has enzymatic activity, a new set of bioassays was performed to test whether the ingestion of formate has any effect on *C. capitata* larvae. For this experiment, 100 mM of ammonium formate was included in the larval artificial diet. As a control, 100 mM of sodium oxalate was also included in the bioassay. In parallel, solubilized OxdD (5 mg per well) with and without oxalate and a whole culture of *B. pumilus* strain 15.1, with and without oxalates were also assayed to determine if the combination of these elements showed any effect on toxicity (Table 3). The presence of oxalate or formate in the diet showed twice the mortality of the water control. However, while no effect on larval size was observed in oxalate bioassays compared to the control, a substantial reduction was noticed when formate was present (larvae did not progress further than first instar), indicating that formate interfered in larval development. When solubilized OxdD (5 mg per well) was included in the diet with or without oxalate, similar mortalities were obtained (around twice that of the water control). No differences in mortality were observed when *B. pumilus* 15.1 strain was assayed either in the presence/absence of oxalate (around three times more mortality than control). These results seem to indicate that the addition of oxalate to the larval diet has no mayor effects on *C. capitata* mortality, either when it was bioassayed alone or together with solubilized crystals/whole *B. pumilus* culture. However, when the formate was present in the diet, larvae were highly undeveloped.

**Table 3:** Mortality results obtained after 10 days in *C. capitata* larvae bioassays using different chemicals (oxalate and formate), *B. pumilus* 15.1, and soluble oxalate decarboxylase. The increase in toxicity compared to the negative control was also calculated

Bioassay	% Mortality	Fold increase
H_2_O (−ve control)	14.35	1
Formate (100 mM)	27.35 ± 3^a^	1.8
Oxalate (100 mM)	29.84 ± 2	2.0
Oxalate (100 mM) + Soluble OxdD^b^	28.3 ± 24	1.9
Soluble OxdD^b^	27.19 ± 6	1.8
*Bp*15.1	41.67 ± 10	2.8
*Bp*15.1 + oxalate (100 mM)	44.79 ± 25	3.0

a The body size of larvae found in this bioassay was similar to first‐instar larvae.

b The amount of soluble oxalate decarboxylase (OxdD) was 5 μg per well (10 μg ml^−1^ of diet).

## Discussion

In this work we have characterized the parasporal crystals of *B. pumilus* strain 15.1 and shown them to consist of a member of the oxalate decarboxylase family of proteins. To our knowledge, this is the first example of a member of an enzyme family found in parasporal crystals.

To establish the location of the gene encoding the parasporal crystals of *B. pumilus* 15.1, both plasmids of the strain (Garcia‐Ramon *et al*., 2015b) were removed. The conventional plasmid curing methods, involving culture at high temperature and/or in the presence of replication‐interfering chemical compounds, have been applied to many bacteria (Hara *et al*., 1982; Ward and Ellar, 1983; Mahillon *et al*., 1988; Sivropoulou *et al*., 2000). Unfortunately, these techniques are not successful in all strains (Rajini Rani and Mahadevan, 1992; Feng *et al*., 2013). In fact, using the most conventional treatments (Ward and Ellar, 1983; Mahillon *et al*., 1988; Ghosh *et al*., 2000; Molnar *et al*., 2003) we were not able to isolate a plasmid‐free variant of *B. pumilus* 15.1. We assayed subinhibitory concentrations of SDS, acridine orange and promethazine combined with high temperature (42°C), but plasmids were not eliminated (data not shown). Based on previous studies, it was proposed that the cell wall/cell membrane could serve as a barrier resulting in inefficient plasmid elimination (Spengler *et al*., 2003). Hence, the curing strategy developed here was based on obtaining spheroplasts of the cells before the treatment with the replication‐interfering compounds. The strategy was highly efficient compared to the conventional methods used for spore‐forming bacteria and was faster, as no successive culturing steps were needed. The method described here could represent a useful approach in those strains resilient to plasmid loss using conventional methods, especially in Gram‐positive bacteria (we note that *B. pumilus* may be tolerant to higher levels of acridine orange than other species and that this sensitivity should be determined before carrying out this step at an appropriate permissive concentration). Our experiments demonstrated that the *oxdD* gene of *B. pumilus* strain 15.1 was located on the chromosome. Although many genes encoding crystals (such as Cry toxins) are encoded by plasmids, there are some encoded in the chromosome (Hu *et al*., 2008; Wang *et al*., 2014). The cured *B. pumilus* strain 15.1C, showed a parasporal crystal production approximately double that of the wild‐type strain. This may indicate that either the small plasmid pBp15.1S or the megaplasmid exerts some kind of direct or indirect regulation on the expression of the *oxdD* gene. Most of the CDSs on these plasmids represent hypothetical proteins but the strain 15.1 genome contig 48, here shown to be part of the megaplasmid in this strain, does appear to encode a YdeB‐like putative transcription factor, an HTH‐type MerR family transcriptional regulator, a potential RNA binding regulator of transcription, that is, Hfq‐like, and a response regulator protein; although no link between these CDSs and OxdD production has yet been established. The megaplasmid also appears to encode the 17 kDa YuaB protein, which has homologues in *B. subtilis* and a hypothetical protein, BPUM_1610 in *B. pumilus* SAFR‐032. In *B. subtilis*, YuaB is a small, secreted protein, that is, localized at the cell wall, plays a role during biofilm formation (Ostrowski *et al*., 2011) and is responsible for forming a layer on the surface of the biofilm making it hydrophobic (Kobayashi and Iwano, 2012). In contrast to *B. pumilus* 15.1, in *B. subtilis* the *yuaB* gene appears to be encoded chromosomally.

Oxalate decarboxylase is a member of the cupin family of proteins, which has enzymatic members but also includes non‐enzymatic proteins including seed storage proteins. The *B. pumilus* 15.1 oxalate decarboxylase, along with storage proteins such as canalvalin and phaseolin, is a bicupin as it has 2‐beta sandwich cupin domains (Tanner *et al*., 2001; Fig. 2) each one containing one manganese binding site (Anand *et al*., 2002). The seed proteins are known to show proteinase resistance, as seen for the protein described here. The protein from *B. pumilus* crystals appears to form a hexameric complex, consistent with the oxalate decarboxylase from *B. subtilis* that in solution (Svedružić *et al*., 2007) and in X‐ray crystallographic analysis (Anand *et al*., 2002) also forms hexamers.

Oxalate decarboxylase (EC 4.1.1.2) catalyses the conversion of oxalate to formate and carbon dioxide. The first bacterial oxalate decarboxylase was identified in *B. subtilis* (OxdC, formerly known as YvrK) as a cytosolic enzyme (Tanner and Bornemann, 2000). Subsequently, a second hypothetical protein (YoaN) from *B. subtilis* exhibited oxalate decarboxylase activity and was named OxdD (Tanner *et al*., 2001), which was found to be present in the interior layer of the spore coat (Costa *et al*., 2004). In *B. subtilis*, OxdC and OxdD are spore‐associated proteins (Kuwana *et al*., 2002), and the recombinant proteins overexpressed in *E. coli* are soluble showing oxalate decarboxylase activity only when expressed in the presence of manganese salts (Tanner *et al*., 2001). We have demonstrated that the accumulation of the *B. pumilus* 15.1 oxalate decarboxylase is dependent on the Mn^2+^ concentration in the medium, consistent with putative promoter elements identified upstream of the gene.

The oxalate decarboxylase crystals were found to solubilize at low temperature (−20°C), a phenomenon that has not previously been described for a crystal protein. This is interesting in the light of the fact that toxicity of the original *B. pumilus* 15.1 strain was dependent on the incubation of the whole culture at low temperature for at least 4 days (Molina *et al*., 2010). In addition, oxalate decarboxylase parasporal crystals purified from *B. pumilus* 15.1 were not significantly toxic in diet contamination assays against *C. capitata* larvae, but a slight increase of toxicity (2–3 times) was observed when solubilized protein was used (Tables 1 and 3).

Although the oxalate decarboxylase protein is not able to induce the mortality of *C. capitata* larvae by itself, we cannot rule out the possibility that this protein may play some role in this process as other virulence factors could be necessary for full toxicity. There are few reports in the literature of oxalate decarboxylase in relation to virulence. The substrate for this enzyme (oxalic acid or oxalate) is associated with several plant pathogenic fungi from the genus *Sclerotinia* (Bateman and Beer, 1965; Kritzman *et al*., 1977; Magro *et al*., 1984). Although the exact mechanism of oxalic acid as a virulence factor is not completely understood, its ability to chelate calcium ions, or to change pH, favouring some cellulolytic enzymes (Lumsden, 1979) or to act as a plant defence inhibitor (Mayer and Harel, 1979; Ferrar and Walker, 1993) seems to help the fungi to invade host plants. Pseudomonad‐like bacterial strains synthesizing oxalate degrading enzymes (Dickman and Mitra, 1992) are reported to prevent *Sclerotinia sclerotiorum* infections in plants by removing the fugal virulence factor oxalate. Oxalate decarboxylase has been used in biological control of fungal plant diseases (Kesarwani *et al*., 2000; Dias *et al*., 2006) making transgenic plants resistant to fungal pathogens.

The fact that the oxalate decarboxylase is overexpressed in *B. pumilus* 15.1 suggests an important role for the bacterium. We have demonstrated that oxalate decarboxylase present in *B. pumilus* 15.1 crystals shows enzymatic activity when solubilized, as formate production was detected in *in vitro* enzymatic assays. The action of oxalate decarboxylase on its only described substrate, oxalate (Brenda database (Schomburg, 2015)), could produce a significant amount of formate when *B. pumilus* 15.1 is bioassayed and this could explain the toxicity of the strain towards *C. capitata* larvae. Formate is well known for being a compound toxic for insects and other arthropods and higher organisms (Elzen *et al*., 2004; Chaskopoulou *et al*., 2009; Underwood and Currie, 2009; Chen *et al*., 2012, 2013), and we have shown it to have a particularly detrimental effect on *C. capitata* larvae development. The origin of the oxalate substrate for the enzyme to produce formate in the environment is not known. The production of oxalate in bacteria is not a very frequent characteristic but in a few cases its production has been related with virulence. This has been demonstrated for *Burkholderia glumae*, a plant pathogen that causes seedling and grain rot via the production of oxalate (Li *et al*., 1999). Although we cannot state definitively whether strain 15.1 is able to produce oxalate, the genome data for this strain (Garcia‐Ramon *et al*., 2015a) do not appear to exhibit genes encoding ascorbate 2,3 dioxygenase (which can produce oxalate from L‐ascorbate), (S)‐hydroxyl acid dehydrogenase (which can produce oxalate from glyoxalate) or oxalate CoA transferase and glyoxylate dehydrogenase (which together can produce oxalate from glyoxylate via oxalylCoA). The genome does, however, encode a putative oxalate:formate symporter in the MSF family, which is present in other *B. pumilus* genomes and is conserved in other bacilli but is found in few species outside this genus, so we could speculate that the strain could utilize oxalate from the medium and use oxalate decarboxylase to produce formate as a virulence factor. However, our data showed that an external supply of oxalate in the larval diet seems not to have any effect on toxicity. Clearly many questions still remain unanswered in the mode of action of *B. pumilus* strain 15.1, but this work represents a step forward in the understanding of this bacterium in relation to putative novel virulence factors that may be used by entomopathogenic bacteria. Characterization of the kinetics of the enzyme and further investigations of its relationship with toxicity will be undertaken in further studies.

## Experimental procedures

### Bacterial strain and growth conditions

The bacterial strain used in this study was *Bacillus pumilus* 15.1 (Molina *et al*., 2010). Luria‐Bertani (LB) medium was routinely used for growing bacteria. When sporulation was required, T3 medium (Travers *et al*., 1987) was used and incubation was at 30°C for 72 h at 240 rpm. Modified T3 medium was also used with different concentrations of MnCl_2_ (ranging from 0 to 0.5 g l^−1^).

### Protein expression profile determination under different conditions

*B. pumilus* 15.1 was grown in 3 ml of LB at 30°C and 240 rpm overnight and used to inoculate 50 ml of T3 medium for growth under the conditions described above for 72 h. Samples (1 ml) were centrifuged for 1 min at 16 000 *g*. Pellets were resuspended in 50 μl of PBS, analysed by SDS‐PAGE and stained with Coomassie brilliant blue, according to standard procedures. Precision Plus Protein™ Standards (Bio‐Rad, Hercules, California, USA) molecular weight marker was used in all SDS‐PAGE gels.

### Discontinuous sucrose gradient

To isolate the parasporal crystals, sporulated cultures (72 h of incubation) grown in T3 medium were subjected to the procedure described by Garcia‐Ramon *et al*. (2016) for discontinuous sucrose gradient separation.

### Protein analysis by 2D gel electrophoresis

Analyses by 2‐dimensional (2D) gel electrophoresis were carried out according to the manufacturer's recommendations (Bio‐Rad). Briefly, 15 μl of each protein sample was mixed with 115 μl of re‐hydratation solution (7 M of urea, 2 M of thiourea, 4% CHAPS, 10 of mM DTT and 0.2% ampholytes) and loaded onto IPG strips (Ready Strip™ IPG Strips 11 cm, pH 3–10, Bio‐Rad). The strips were re‐hydrated at 20°C for 16 h (passive rehydration) in a Protean^®^ IEF Cell (Bio‐Rad). Isoelectric focusing (IEF) was carried out using the following four‐step programme: (i) 250 V for 1 h in a linear mode, (ii) 4000 V for 2 h in a linear mode; (iii) 4000 V until 18 000 Vh in a rapid mode; 500 V until 50 μA per strip in a rapid mode. After IEF, strips were equilibrated for 10 min in equilibration buffer I (6 M urea, 0.375 M Tris‐HCl pH 8.8, 2% SDS (wt/vol), 20% glycerol (vol/vol)) containing 130 mM DTT, followed by an incubation in equilibration buffer II, containing 135 mM of iodoacetamide instead of DTT, for 10 min. Proteins were then separated by their molecular weight by placing the strip on the top of a 12% SDS‐PAGE in a vertical electrophoretic unit (Bio‐Rad). Electrophoresis was performed at 120 V for 60 min. Two‐dimensional gels were stained with Coomassie blue.

### Solubilization of crystals and protease treatment

Fractions from a discontinuous sucrose gradient containing most of the crystals produced by *B. pumilus* 15.1 were kept frozen at −20°C until use. To determine protease stability of the 45 kDa protein, the sample was thawed on ice and centrifuged at 13 000 rpm for 3 min, and the supernatant was collected in a fresh tube. Protein concentration was determined in the supernatant using Bradford's reagent (Sigma, St Louis, Missouri, USA), following the manufacturer's recommendations and using bovine serum albumin BSA (Sigma) as a standard. Supernatant fractions were incubated with four different proteolytic enzymes: trypsin, chymotrypsin, papain and ‘Proteinase from *Bacillus subtilis’* (cat No. 96887) from Sigma. Buffers and incubation temperatures for each enzyme were chosen according the instructions provided by the supplier. The standard ratio used for protease treatment was 10:1 (w/w; protein:protease), although other ratios were tested. Samples were incubated for 1 h, and a BSA control was carried out in parallel to verify protease activity. A sample without proteases was also incubated under the same conditions as a negative control. For comparative purposes, the solubilized Cry1Aa13 (expressed in *Escherichia coli* from plasmid pCP10 (Pigott, 2006) was also digested at the same protein:trypsin ratios (between 1:1 to 1:500, protein:trypsin). All the digested proteins were analysed by SDS‐PAGE.

### Transmission electron microscopy

Fresh aliquots from the sucrose gradient fractions were pelleted and washed following the methodology previously described (Garcia‐Ramon *et al*., 2016) and sent to the ‘Biological Sample Preparation Laboratory’ at the Scientific Instrumentation Center of the University of Granada (CIC‐UGR) for processing. Samples were observed under a Transmission Electronic Microscope (LIBRA 120 PLUS from Carl Zeiss SMT, Oberkochen, Germany) in the Microscopy Service of the CIC‐UGR. Ten images of 12.6 μm in size were used to determine the crystal:spore ratio.

### Plasmid curing procedures

Three procedures reported in the literature were tested for the curing of the extrachromosomal elements present in the strain *B. pumilus* 15.1. In the first place, the methods described by Ward and Ellar (1983) and Mahillon *et al*. (1988), based on culturing the strain at high temperature were used with slight modifications. *B. pumilus* strain 15.1 was grown in 3 ml of LB for 24 h at 42°C and 240 rpm. Successive dilutions of the culture (1:100) into fresh medium were made after 12 h of incubation during a total period of 72 h. The second method tested was performed as described above, with the difference that LB medium was supplemented with 0.002% SDS (Sivropoulou *et al*., 2000). In the third procedure, the *B. pumilus* 15.1 strain was grown in LB supplemented with 0.03% acridine orange or 0.12% promethazine for 24 h, either at 30°C or at 42°C. Bacterial cultures were transferred (1:100 dilution) into fresh LB medium supplemented with the interfering compounds every 12 h for 5 days.

Cells derived from these procedures were plated on LB medium and incubated for 12–24 h at 30°C. Randomly selected colonies were used for total DNA extraction using the methodology described by Reyes‐Ramirez and Ibarra (2008). Total DNA was analysed by electrophoresis in a 0.8% (wt/vol) agarose gel with SYBR Green from Invitrogen.

In addition to the standard methods, above, we also developed a novel curing strategy. For this, *B. pumilus* 15.1 was cultured in 5 ml of LB medium to an optical density at 600 nm of 0.9 to 1.1. One millilitre of the culture was pelleted at 16 000 *g* for 1 min. The pellet was resuspended in 1 ml of PBS containing 2% (wt/vol) lysozyme and 20% (wt/vol) sucrose and was incubated at 37°C for 90 min. In this period of time, more than 90% spheroplast formation was achieved as monitored under the microscope. The spheroplast suspension was diluted 1:100 in LB medium supplemented with 0.03% acridine orange or 0.12% promethazine and cultured at 30°C and 240 rpm for 48 h until growth was observed. Serial dilutions were plated on LB plates and incubated at 30°C overnight.

### Plasmid copy number determination

Plasmid copy number was determined by quantitative real‐time PCR as previously described (Garcia‐Ramon *et al*., 2015b). Briefly, total DNA was used to amplify the *smc* gene, that is, present in a single copy on the chromosome with smc_F and smc_R primers and orf7_F and orf7_R primers were used to amplify a unique region in the pBp15.1S plasmid.

### Southern blot analysis

Total DNA was electrophoresed on a 0.8% (wt/vol) agarose gel and stained with ethidium bromide and transferred to a nylon membrane. The PCR product (855 ng) amplified with orf7_F and orf7_R primers (Garcia‐Ramon *et al*., 2015b) and cleaned with QIAquick^®^ PCR Purification kit (Qiagen, Hilden, Germany) were used as a probe for the pBp15.1S plasmid. DNA labelling, transfer and fixation to the membrane, hybridization and immunological detections were performed with a DIG DNA Labeling and Detection Kit (Roche No. 11093657910, Basilea, Switzerland) following the instructions provided by the supplier.

### Mass spectrometric analysis of protein samples

Bands or spots identified for analysis from the 1D or 2D SDS‐PAGE gels were individually excised and sent to ‘Centro de Investigación Principe Felipe’, Valencia‐Spain, for the peptide identification by Matrix‐Assisted Laser Desorption Ionization‐Time Of Flight Mass Spectrometry (MALDI TOF‐MS). Digestion products were analysed by MALDI MS (4700 Proteomics analyser of the Applied Biosystems, Foster City, California, USA). Searches of the *B. pumilus* 15.1 genome (Garcia‐Ramon *et al*., 2015a) and public databases were performed using MASCOT search engine (Matrix‐Science, London, UK). The services from ‘SCSIE University of Valencia Proteomics Unit’ and ‘CBMSO Protein Chemistry Facility’ that belong to the ProteoRed Proteomics Platform were also used. At the SCSIE University of Valencia Proteomics Unit a MALDI‐TOF MS/MS analysis (5800 MALDI TOFTOF ABSciex) was performed. The MS and MS/MS information was analysed by MASCOT via the Protein Pilot (ABSciex, Framingham, Massachusetts, USA). Database search was performed on NCBInr.

At the CBMSO Protein Chemistry Facility (Madrid) a Liquid chromatography tandem mass spectrometry (LC‐MS/MS) analysis (Orbitrap‐LTQ‐Velos‐Pro, Thermo Scientific, Waltham, Massachusetts, USA) was performed, and the search was made on UniProt‐*Bacillus* and UniProt‐*Bacillus pumilus* databases, using proteome discoverer 1.4 software (Thermo Scientific, Waltham, Waltham, Massachusetts, USA).

### N‐terminal amino acid sequencing

The solubilized and trypsinized protein of 45 kDa was separated in a 12% acrylamide SDS PAGE gel with Tris Tricine running buffer. Separated proteins were blotted onto PVDF membrane using a semi‐dry transfer blotter. N‐terminal sequencing was performed by Abingdon Health Laboratory Services, Birmingham, UK.

The sequence obtained was compared with protein sequences from the genome of *B. pumilus* 15.1 (GenBank LBDK00000000.1; Garcia‐Ramon *et al*., 2015a).

### Size‐exclusion chromatography

Soluble oxalate decarboxylase protein from *B. pumilus* strain 15.1 was analysed by size‐exclusion chromatography using a HiLoad 16/600 Superdex 200 prepacked column (GE Healthcare, Life Science, Chicago, Illinois, USA) in 50 mM of sodium phosphate (pH 5.0), 300 mM of NaCl using an AKTAPure 25 system (GE Healthcare). The molecular weight of oxalate decarboxylase in solution was determined by reference to a calibration curve obtained on the same column with gel filtration standards (Bio‐Rad).

### Primer design and PCR amplification of the hypothetical protein YuaB

To PCR amplify the *yuaB* gene, the primers YuabF (5′ AAAAAGATCTAACCAAATGCGCTATTCCCC 3′) and YuabR (5′ AAGAATTCCTTTGTCAACAATCTGAAGCGC 3′) were designed based on the sequence from *B. pumilus* 15.1 (Garcia‐Ramon *et al*., 2015a). Total DNA from the wild type and the cured strain was used under the following PCR conditions: 95°C for 5 min, followed by 30 cycles of 95°C for 1 min, 55°C for 1 min and 72°C for 1 min and then a final extension at 72°C for 5 min. Amplification was checked by electrophoresis on a 1% (wt/vol) agarose gel.

### *C. capitata* larval bioassays

Bioassays with *B. pumilus* strain 15.1 were performed as described previously (Molina *et al*., 2010). When ammonium formate or sodium oxalate was bioassayed, solid powder from these compounds was dissolved in the diet to a final concentration of 100 mM. The insecticidal activity of insoluble parasporal inclusion suspensions obtained from *B. pumilus* 15.1 was tested at a cell density approximately 40 times greater than the original culture following Molina *et al*. (2010) with some modifications. When solubilized, oxalate decarboxylase was assayed at 10 μg ml^−1^ of diet (5 μg per well). Briefly, 100 μl of the sample was dispensed into each well and mixed with 500 μl of artificial diet. One larva of *C. capitata* was placed in each well. The bioassays were performed in 48‐well sterile Cellstar microplates (Greiner Bio‐one, Kremsmünster, Austria) at 25°C. Deionized water was used as negative control. All bioassays were performed at least twice using different cultures or crystal samples obtained from separate cultures and gradients. In all bioassays, mortality was recorded 10 days after the beginning of the bioassay.

### Enzymatic assays

The activity of oxalate decarboxylase was evaluated by the production of formate using two methods. In the first, the oxalate decarboxylase activity assay kit (Sigma Aldrich) was used according to the manufacturer's instructions. Results were compared to a range of concentrations of formate and with the activity of an oxalate carboxylase positive control (both provided in the kit). The second assay detected formate production by nmr. Briefly, 300 μl of sodium phosphate buffer (100 mM, pH 5.0) was mixed with 200 μl of sodium oxalate (300 mM, pH 5.0) in a final volume of 600 μl containing 0, 5 or 10 μg of oxalate decarboxylase enzyme (previously purified by sucrose gradient and solubilized in Milli Q water at low temperature as described above). When indicated, 1 mM MnCl_2_ was included in the assay. The mixture was incubated for 2 h at 37°C, and the reaction was stopped with 1 ml of sodium phosphate buffer (150 mM, pH 9.5). Then, methanol (reagent grade, Sharlau) was added to each sample to a final concentration of 5 mM as an internal reference for ^1^H‐NMR analysis. Samples were analysed in a Varian Direct Drive Spectrometer of 500 MHz at the Centro de Instrumentación Científica of the University of Granada. Spectra were obtained under fully relaxed conditions, and the water signal was suppressed. The area of each peak was integrated using mestrenova 9.0 software (Mestrelab Research, Santiago de Compostela, Spain) taking the methanol signal as an internal reference.

## Conflict of interest

All authors declare there is no conflict of interest.

## Supporting information


**Fig. S1.** Multimeric form of *B. pumilus* 15.1 Oxalate decarboxylase determined by size‐exclusion chromatography.

**Fig. S2.** Representative spectra obtained in the H‐NMR analysis.
