# Supplementary figures and images for: The parasporal crystals of Bacillus pumilus strain 15.1: a potential virulence factor?
Source: Microb Biotechnol. 2017 Oct 12;11(2):302–16. doi: 10.1111/1751-7915.12771 (PMC5812249; doi:10.1111/1751-7915.12771)

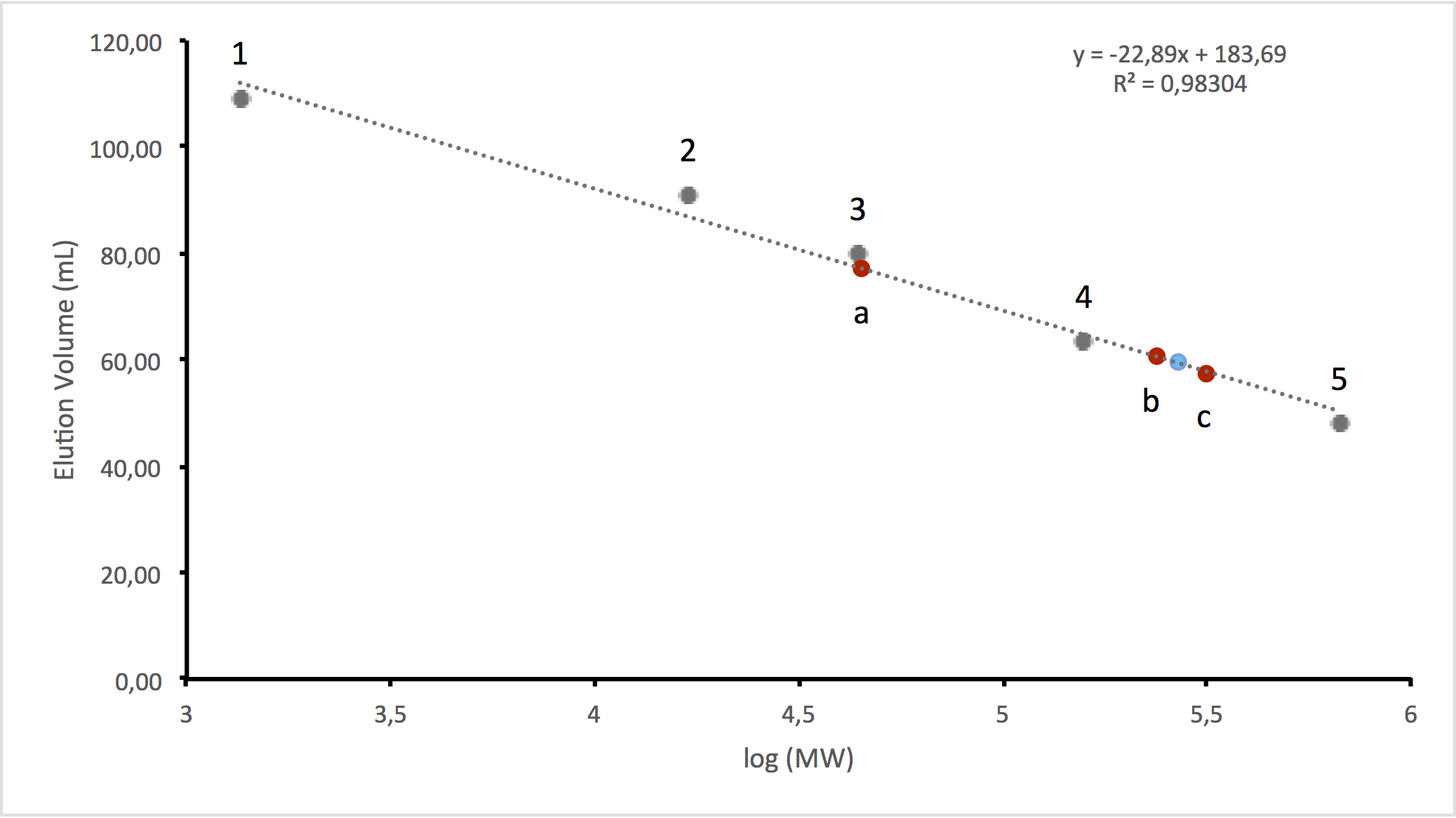

Supplement: Supplementary file 1 — Fig. S1. Multimeric form of B. pumilus 15.1 Oxalate decarboxylase determined by size‐exclusion chromatography. [file MBT2-11-302-s001.tiff]

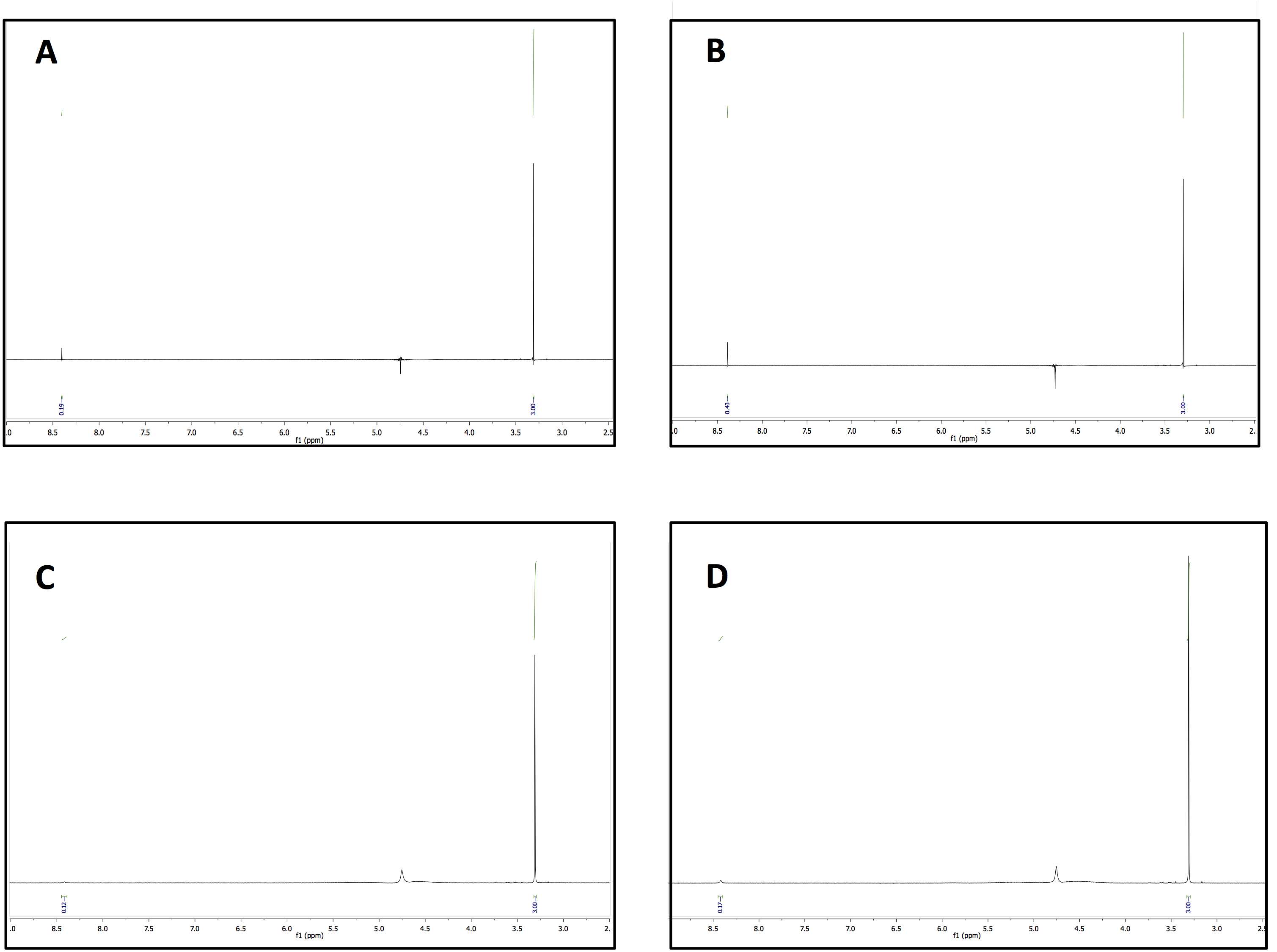

Supplement: Supplementary file 2 — Fig. S2. Representative spectra obtained in the H‐NMR analysis. [file MBT2-11-302-s002.tiff]
